# 

**Published:** 1899-11-25

**Authors:** 


### NOTICE TO CORRESPONDENTS.

All MS., Letters, Books for Review, and other matters intended for the Editor should be addressed The Editor, "The Hospital," The Hospital Building, 28 & 29, Southampton Street, Strand, W.C.

The Editor cannot undertake to return rejected MS., even when accompanied by stamped directed envelope.

**Notice to Advertisers and Subscribers.**

All Advertisements, Orders for Copies of The Hospital, and Business Communications should be addressed to **THE MANAGER (Not to the Editor)**, The Hospital, The Hospital Building, 28 & 29, Southampton Street, Strand, London, W.C.

The Cost of Subscription, post free, is as follows:—Per week, 2½d.; per quarter, 2s. 8d.; per half-year, 5s. 3d.; per annum, 10s. 6d. Foreign:—Per annum, 15s. 2d., payable, in all cases, in advance.

**NOTICE.—Vol. XXVI. of "The Hospital" (April, 1899, to September, 1899), in handsome cloth binding, is now on sale at the Office of this Journal, price 7s. 6d. Cases for binding the Numbers contained in this Volume 1s. 6d. Index to Vol. XXVI., 2½d., post free.**

**The Monthly Part for December will be ready on December 1st. Price 6d., by post 8d.**

### BOOKS RECEIVED.

Simpkin, Marshall, and Co.

"The Maintenance of Health in Egypt." By Arthur J. M. Bentley, M.D.

J. and A. Churchill.

"The Pathologist's Handbook: A Manual for the Post-mortem Room." By T. N. Kelynack, M.D., M.R.C.P.

D. Fourdrinier, "Builder" Office.

"Architectural Hygiene; or, Sanitary Science as Applied to Buildings." By Banister F. Fletcher and H. Phillips Fletcher.

Longmans, Green, and Co.

"Surgery: A Treatise for Students and Practitioners." By Thomas Pickering Pick.

### Scraps and Gleanings.

It is anticipated that there will be a deficit on this year's accounts of the Newcastle Royal Infirmary of £3,000.

It is estimated that the cost of the proposed extensions at Oldham Infirmary will amount to about £16,000, half of which amount has been already promised.

At a recent meeting of the Executive Committee of the Cardiff Infirmary it was resolved that in future no charge be made for supplying medical certificates when applied for by patients treated at the infirmary.

Since the foundation in 1834 of the Dublin Hospital Sunday Fund the total amount of contributions has been upwards of £103,000. It is found that the hospitals aided by the Sunday Fund draw 8 per cent. of their income from this source.

The number of in-patients treated during the past year at the Bridgwater Infirmary was 355, compared with 350 the previous year. The number of out-patients has largely increased, viz., from 1,574 for the year ending August 31st, 1898, to 2,079 for the corresponding period this year. There has been a considerable increase in the number of surgical cases, the number of operations performed being 152, while last year it was only 86.

Since the Ockenden Convalescent Home was opened in 1890 over 1,000 persons have been received into it. It has recently been thoroughly renovated at a cost of £400 through the generosity of the sister of the late founder, Miss Erle.

About £600 is needed by the Portsmouth Eye and Ear Infirmary to free the committee from all anxiety as to expense in connection with the new buildings which are now completed, and which provide accommodation for double the number of patients previously accommodated.

A sub-committee is to reconsider the plans of the proposed joint infectious hospital for Dewsbury and submit a scheme for the provision of the required accommodation at a cost not exceeding, if possible, £22,000. It has also been decided to consult the medical officer of health.. War Office for their Swiss milk for use of the troops in South Africa.

The total amount of this year's Hospital Sunday Fund collection at Liverpool shows a decrease from last year's, viz., £6,177 odd this year as against £6,215 odd in 1898. The Saturday Fund, however, shows an increase, so that the joint committee of these two funds have been able to make slightly increased grants this year to the participating institutions, and in all have distributed an amount of £12,611.

### The Student of Medicine.

### APPOINTMENTS.

J. Baker, M.B., C.M.Aberd., appointed Deputy Superintendent, State Criminal Lunatic Asylum, Broadmoor. E. M. Brockbank, M.D.Vict., M.R.C.P.Lond., appointed Honorary Physician to the Ancoats Hospital, Manchester. W. d'E. Emery, M.D., B.Sc.Lond., M.R.C.S., L.R.C.P., appointed Assistant Surgeon to the Birmingham and Midland Skin and Urinary Hospital. E. L. Forward, M.R.C.S.Eng., L.R.C.P.Lond., appointed Assistant Medical Officer to the Coppice Asylum, Nottingham. W. P. Montgomery, M.A., B.S. & M.B.Lond., F.R.C.S., appointed Honorary Surgeon to the Ancoats Hospital, Manchester. C. T. Parsons, M.D.Lond., appointed Medical Superintendent to the Fulham Infirmary. I. B. Richardson, M.R.C.S., L.R C.P.Lond., appointed Assistant House Physician for the General Hospital, Birmingham. S. Savage, M.A., M.B., B.A.Oxon., F.R.C.S Eng., appointed Honorary Surgeon to the Birmingham Lying-in-Charity. M. F. Squire, M.B.Durh., and B.S., appointed Medical Superintendent of the Infirmary and Medical Officer of the Workhouse of the Parish of Paddington. F. H. Westmacott, F R.C S., appointed Honorary Aural Surgeon to the Manchester Children's Hospital, Pendlebury. D. J. Young, M.B., Ch.B.Glasg., appointed Casualty Surgeon of the St. Rollox Division of Police, Glasgow. G. N. Meachen, M.B. Lond., M.R.C.S., L.R.C.P., has been appointed House Surgeon to the Tottenham Hospital, N.

### A NEW TEXT-BOOK FOR STUDENTS.

Crown 8vo., nearly 150 pp. Illustrated. Cloth. Price 2s. 6d.

**MEDICAL GYMNASTICS,** Including the SCHOTT (Nauheim) Movements. Being a Text-Book of Massage and Mechanical Therapeutics Generally, for Medical Students. By AXEL V. GRAFSTROM, B.Sc., M.D. (Late Lieutenant in the Royal Swedish Army; late House Physician, City Hospital, Blackwell's Island, New York.)

London: The Scientific Press, Ltd., 28 & 29, Southampton Street, Strand, W.C.

**THE UNIFORM S[illegible]STEM OF ACCOUNTS, AUDIT, AND TEND[illegible]RS, for Hospitals and Institutions, with Certain Ch[illegible]cks upon Expenditure; also the Index of Classificatio[illegible]** Compiled by a Committee of Hospital Secretaries, and adopted [illegible], a General Meeting of the same, 18th January, 1892. And cert[illegible] Tender and other Forms for securing Economy, By Sir HENR[illegible] BURDETT, K.C.B. Demy 8vo, profusely illustrated with Specimen Tables, Index of Classification, Forms of Tender, &c., cloth extra, 6s.

---

**ACCOUNT BOOKS FOR INSTITUTIONS, designed in accordance with the Uniform System of Accounts for Hospitals and Institutions.** By Sir Henry Burdett, K.C.B. Being a complete set of Account Books ruled in accordance with the Uniform System adopted by the Metropolitan Hospital Sunday Fund.

*(Full Description of Books and Prices sent post free on application.)*

London: The Scientific Press, Ltd., 28 & 29, Southampton Street, Strand, W.C.

Accouchement Requisites.

Ambulance Cases.

### NOW READY,

A REPRINT OF

### A NURSING HANDBOOK

TOGETHER WITH S. MAW, SON, AND THOMPSON'S

### COMPLETE CATALOGUE OF NURSING REQUISITES.

The above work is now republished at a cost of 1s. per copy. Messrs. S. M., S., and T. have been compelled to make this charge on account of the extreme popularity of the work and the great expense incurred in its production.

***Post Free on receipt of 1s.***

### S. MAW, SON, and THOMPSON,

7 to 12, ALDERSGATE STREET, LONDON, E.C.

